# Operations by Students

**Published:** 1871

**Authors:** 


### ARTICLE IV.—*Operations by Students.*

The examiners and teachers of the rising generation of surgeons have lately become awake to the strange inconsistency of its not having been necessary for candidates for surgical diplomas to prove themselves surgeons in fact. Of the vast numbers of men who have, up to the present time, withdrawn from hospital training into private practice, the majority had not even undergone a complete course of instruction in operative surgery on the dead body, and comparatively few ever used the knife for any other purpose than dissection until the health and lives of patients began to depend as much on their coolness of judgment and skill of hand as on their theoretic knowledge.

We therefore note, with much satisfaction, an incident of some novelty, which is mentioned in one of our hospital reports of this week, as having occurred in the operating theater of one of the principal London hospitals—namely, the performance of an operation by a dresser, in the presence of his fellow-students, and under the immediate eye of his teacher. We understand that it is the intention of this gentleman to accord to those of his dressers, whose diligence and skill merit recognition, the privilege and skill of performing, during their term of office, a few simple operations in the hospital theatre, and have little doubt that this liberal experiment will prove to be not only a great incentive to diligent and careful study, but the best possible means of cultivating those qualities which are peculiarly necessary to the composition of a good surgeon.—*Lancet.*

